# Catecholaminergic Adaptation to Extreme Military Stress: Norepinephrine and Dopamine Responses During and After SERE Training

**DOI:** 10.3390/ijms262211012

**Published:** 2025-11-14

**Authors:** Michalina Grzesik-Pietrasiewicz, Kornelia Łach, Krzysztof Przednowek, Rafał Podgórski

**Affiliations:** 1 Department of Medicinal Chemistry and Metabolomics, Faculty of Medicine, Medical College of Rzeszów University, 35-959 Rzeszów, Poland; migrzesik@ur.edu.pl (M.G.-P.); kolach@ur.edu.pl (K.Ł.); 2 Faculty of Physical Culture Sciences, Medical College of Rzeszów University, 35-959 Rzeszów, Poland; krprzednowek@ur.edu.pl

**Keywords:** norepinephrine, dopamine, stress adaptation, SERE, catecholamines

## Abstract

Catecholamines are fundamental mediators of the stress response, regulating arousal, vigilance, and adaptive behavior. However, their dynamics under extreme real-life conditions remain insufficiently explored. Survival, Evasion, Resistance, and Escape (SERE) training provides a unique model for examining neuroendocrine mechanisms of adaptation during both the acute phase and the recovery period following intense psychological and physical stress. Serum norepinephrine (NE) and dopamine (DA) were measured in 47 special forces soldiers during peak stress in SERE and one month later, compared with 17 healthy controls. Samples were collected under standardized conditions and analyzed using validated ELISA kits. NE levels differed significantly among groups (*p* = 0.003), being higher during SERE training and in controls compared to the post-recovery condition. DA also showed a significant group effect (*p* < 0.001), with increased levels during recovery and in soldiers during SERE relative to controls. The post-recovery decline in norepinephrine suggests adaptive habituation of sympathetic activity following extreme stress exposure. Conversely, the sustained elevation of dopamine during recovery may reflect neuroadaptive mechanisms that promote motivational and cognitive restoration. Together, these findings indicate coordinated catecholaminergic regulation supporting long-term resilience in elite military personnel.

## 1. Introduction

Stress is a fundamental adaptive mechanism, enabling organisms to respond rapidly to environmental threats and restore homeostasis. The physiological stress response is orchestrated primarily by two interconnected systems: the sympathetic-adrenomedullary (SAM) axis, which releases catecholamines, and the hypothalamic–pituitary–adrenal (HPA) axis, which regulates glucocorticoid secretion [1,2]. Catecholamines such as norepinephrine (NE) and dopamine (DA) play complementary roles in modulating vigilance, arousal, cardiovascular control, and motivational processes during stress exposure. Acute increases in NE promote rapid mobilization of energy resources and heightened alertness, while DA regulates goal-directed behavior and reinforcement learning. Together, these hormones support adaptive functioning under demanding conditions [3,4,5].

While acute activation of catecholaminergic pathways is essential for survival, chronic or repeated stress exposure may induce maladaptive changes, often described as “allostatic load” [6,7]. Dysregulated NE signaling has been implicated in anxiety, cardiovascular pathology, and post-traumatic stress disorder [8], whereas impaired dopaminergic regulation is linked to reduced motivation, anhedonia, and cognitive inflexibility [9]. However, not all changes are detrimental. Evidence suggests that repeated stress can lead to habituation of adrenergic responses, thereby preventing sympathetic overactivation [10,11], and may also trigger dopaminergic plasticity that enhances recovery, motivational re-engagement, and resilience [12]. These neuroadaptive processes highlight the importance of studying both acute and delayed hormonal responses to stress.

Special forces soldiers are exposed to extreme physical and psychological stressors that demand exceptional resilience and adaptability. Intensive trainings and operational demands may approach or surpass the threshold of an individual’s performance and adaptive capacity. The capacity of soldiers to endure training and operational stressors over time significantly influences their long-term resilience and overall health [13,14]. Realistic simulations of demanding military training provide valuable insight into the levels of stress soldiers may face in both instructional and operational contexts. One such model is the Survival, Evasion, Resistance, and Escape (SERE) program.

SERE training is a rigorous program designed to prepare military personnel for the challenges of isolation, captivity, and survival in hostile environments. SERE prepares specially selected soldiers for the worst-case scenario—isolation behind enemy lines and possible captivity. Its primary objective is to survive in isolation and prolong the window for rescue by allied forces. It does not teach offensive tactics; rather, it ensures personnel can return with honor while upholding legal and ethical standards. This program combines multiple stressors—sleep deprivation, caloric restriction, physical exertion, and psychological pressure—that closely simulate the demands of real-world combat operations [15]. Consequently, the SERE course offers a unique setting to measure physiological stress responses in a highly stressful training environment and is a unique opportunity for study. Most previous studies in this context has focused on immediate stress responses, whereas the delayed regulation of neurochemical systems during recovery remains poorly understood [16,17,18,19].

The present study was undertaken to extend this knowledge by assessing serum norepinephrine and dopamine levels in special forces soldiers both during peak SERE-induced stress and after a one-month recovery period, compared with healthy controls. We hypothesized that norepinephrine would be elevated during acute stress exposure and decline after recovery, whereas dopamine might exhibit a delayed and sustained increase, reflecting neuroplastic adaptations. Our findings of a modest but measurable elevation of dopamine following recovery highlight the importance of considering extended post-stress dynamics in understanding catecholaminergic contributions to resilience.

## 2. Results

**Figure 1:**
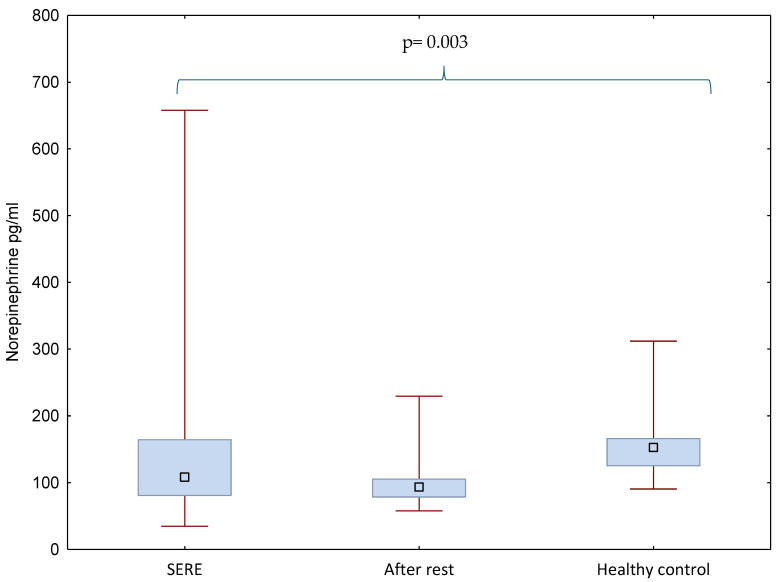
Norepinephrine levels in soldiers (during SERE training and one month later, after a rest period) and in healthy controls; differences between means were analyzed using the Kruskal–Wallis test. The blue box represents the interquartile range (from the first quartile at the bottom to the third quartile at the top), with the median shown inside the box. The lower whisker extends to the minimum value in the data set, and the upper whisker extends to the maximum value.

**Figure 2:**
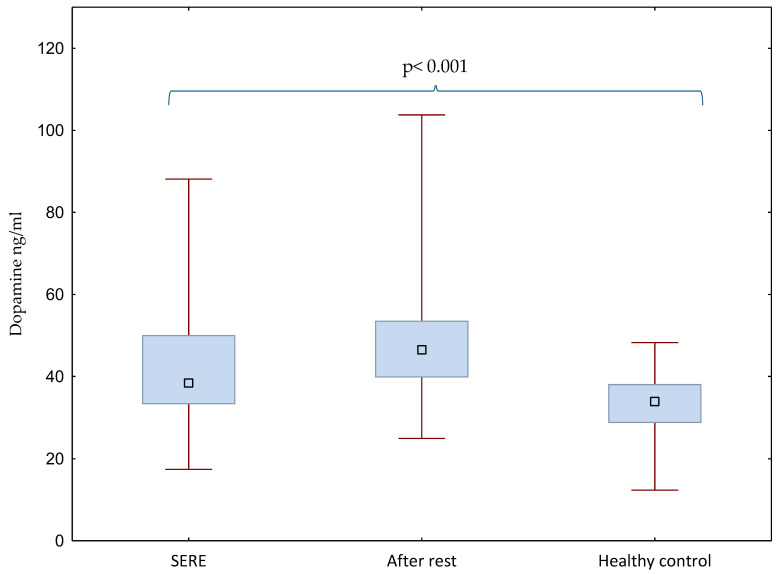
Dopamine levels in soldiers (during SERE training and one month later, after a rest period) and in healthy controls; differences between means were analyzed using the Kruskal–Wallis test. The blue box represents the interquartile range (from the first quartile at the bottom to the third quartile at the top), with the median shown inside the box. The lower whisker extends to the minimum value in the data set, and the upper whisker extends to the maximum value.

The present analyses focused on catecholaminergic markers, norepinephrine and dopamine, measured in 47 special forces soldiers during SERE training, after a one-month recovery period, and in 17 healthy controls. Hormone concentrations and group differences are illustrated in Figure 1 and Figure 2 and the detailed raw data are presented in Supplementary Files S1 and S2.

Norepinephrine levels differed significantly between groups (*p* = 0.003). Soldiers during SERE training had significantly higher and more variable norepinephrine concentrations (155.33 ± 124.83 pg/mL) compared with the post-rest condition (102.32 ± 39.70 pg/mL). Healthy controls (161.81 ± 62.11 pg/mL) also showed significantly higher levels than the post-rest group (Figure 1). Post hoc analysis confirmed significant differences between healthy controls and the after-rest condition (*p* = 0.003).

Dopamine levels also differed significantly between groups (*p* < 0.001). Soldiers after the rest period exhibited higher dopamine concentrations (48.02 ± 14.91 ng/mL) compared with the SERE condition (41.16 ± 13.77 ng/mL). Both SERE and after-rest groups had significantly elevated dopamine levels compared with healthy controls (32.03 ± 10.30 ng/mL) (Figure 2). Post hoc analysis confirmed significant differences between healthy controls and the after-rest condition (*p* < 0.001).

The Wilcoxon signed-rank test did not reveal any significant within-subject differences between soldiers during SERE training and after the rest period, with no differences observed for either hormone.

No significant correlations were found between norepinephrine and dopamine concentrations (Spearman’s rank correlation test, *p* > 0.05).

## 3. Discussion

In this study, we investigated catecholaminergic responses by comparing special forces soldiers during the acute stress of SERE training, after a 28-day recovery period, and a healthy control group. We found that norepinephrine concentrations were elevated during acute stress compared with the recovery phase, while dopamine showed a modest but measurable increase after 28 days of rest, surpassing both acute SERE and control values. These findings expand the existing literature, which has predominantly examined acute stress responses in SERE, with limited data available on long-term neuroendocrine recovery.

Previous work has consistently reported marked increases in stress hormones during captivity and interrogation phases of SERE. For example, Lieberman et al. demonstrated significant elevations in NE, epinephrine, and neuropeptide Y alongside impaired mood and cognitive performance during mock captivity [17]. Similarly, Szivak et al. observed significant increases in both NE and DA during SERE training, with incomplete normalization even after 24 h of recovery, highlighting the short-term persistence of catecholaminergic activation [16]. Studies of winter SERE training, which combine prolonged field activity, caloric restriction, and simulated captivity, have also documented elevated catecholamine concentrations, with only partial recovery after 36 h of rest [20]. Moreover, a recent study aimed at evaluating physiological recovery and metabolic restoration up to 45 days after winter SERE training reported decreased NE concentrations, which may reflect the restoration of energy balance and attenuation of sympathetic drive following prolonged caloric deficit. The authors emphasized the potential influence of nutritional factors on these observations and noted the difficulty in clearly separating the effects of stress from those of diet, as protein intake was intentionally increased during the recovery phase [21]. Our results are consistent with this evidence in demonstrating elevated NE under acute stress but provide a novel contribution by extending observations to a 28-day recovery period, during which soldiers had returned to their regular routines. The significant decline in NE one month after SERE suggests adaptive attenuation of sympathetic activation once soldiers resumed routine duties. This extended recovery period is rarely examined in prior research, which has typically focused on immediate or short-term (24–36 h) recovery. Furthermore, the observed increase in DA after 28 days contrasts with the short-term pattern described by Szivak et al., possibly reflecting delayed dopaminergic upregulation associated with motivational and cognitive recovery processes.

Although norepinephrine has been widely examined as a key marker of the stress response, the dopaminergic response to extreme psychophysiological strain, such as SERE training, remains less well characterized. In the present study, dopamine concentrations were significantly higher during acute stress compared with healthy controls and exhibited a modest yet persistent increase after 28 days of recovery. This pattern suggests a delayed adaptive modulation of dopaminergic activity rather than a transient stress-induced surge. Such a response may reflect compensatory neuroplastic mechanisms involved in motivational re-engagement, reinforcement learning, and the restoration of executive control once acute stress subsides [22,23]. Neurobiological evidence indicates that stress induces region-specific alterations in dopaminergic transmission—transient suppression within the prefrontal cortex accompanied by enhanced mesolimbic signaling, which may promote stress resilience and reward-driven behavior [24]. Human molecular imaging also shows that acute psychosocial stress elicits striatal dopamine release and engages prefrontal dopaminergic signaling, with intensity partially tracking the cortisol response [25,26]. In animal studies, moderate or repeated stress has been associated with elevated dopaminergic tone and improved cognitive flexibility, whereas chronic or uncontrollable stress leads to dopaminergic blunting and motivational deficits [27,28]. Such findings, together with the “inverted U” model of dopamine effects on working memory and cognitive control, support the interpretation that the slight but sustained increase in dopamine observed after 28 days may represent an adaptive recalibration of the mesocorticolimbic pathway, supporting the recovery of motivation, cognitive performance, and affective balance following the cessation of extreme stress [29]. To our knowledge, this is the first study to demonstrate a sustained post-stress elevation in dopamine following SERE training, suggesting that dopaminergic plasticity may contribute to long-term neurobiological adaptation and resilience among special forces soldiers.

Beyond catecholamines, SERE studies have repeatedly identified neuropeptide Y (NPY) and the ratio of dehydroepiandrosterone sulfate (DHEA-S)/cortisol as biomarkers of stress adaptation and performance [30,31]. Higher NPY has been associated with better cognitive/operational performance under captivity-related stress, and more favorable DHEA S–cortisol profiles with reduced dissociation and better outcomes. Situating our catecholamine findings with these markers suggests that lower NE levels upon recovery in our soldiers may indicate a broader ‘resilience phenotype’ characterized by tempered sympathetic tone plus endocrine profiles previously linked to superior performance under extreme stress. These multi-marker associations may help explain why elite operators show rapid functional recovery after SERE.

The physiological stress response represents a complex, multi-axis process involving the hypothalamic–pituitary–adrenal (HPA) axis, the sympathetic–adrenomedullary (SAM) system, and monoaminergic neurotransmission. These systems interact dynamically to support both immediate survival and long-term adaptation. Norepinephrine plays a central role in rapid autonomic activation—mobilizing metabolic resources, enhancing vigilance, and maintaining cardiovascular control—whereas dopamine contributes to motivational drive, cognitive flexibility, and reward-based learning [24,32]. Following the cessation of acute stress, regulatory dominance gradually transitions from noradrenergic to dopaminergic signaling, marking a shift from mobilization to recovery processes. In the present study, the observed decrease in NE and the modest yet sustained elevation in DA after 28 days may reflect a coordinated recalibration of these systems, facilitating the transition from sympathetic arousal toward the restoration of executive control and emotional stability. This pattern is consistent with the concept of allostatic adaptation, wherein neuroendocrine systems readjust to maintain homeostasis through change [33,34,35]. Moreover, dopaminergic plasticity within the mesocorticolimbic pathway may play a crucial role in reinforcing motivational re-engagement and resilience following extreme stress exposure [36,37]. The absence of a significant correlation between norepinephrine and dopamine concentrations further supports the notion that these catecholaminergic systems function through partially distinct yet complementary regulatory mechanisms during and after severe psychophysiological strain.

In summary, the obtained results indicate dynamic and adaptive changes within the catecholaminergic system that may support the recovery of cognitive and emotional functions following extreme stress. Understanding these processes not only provides insight into the neurobiological mechanisms of resilience but may also inform the design of more effective strategies to enhance psychophysiological restoration and operational readiness in military personnel exposed to high-intensity stress.

Nevertheless, several limitations of the present study should be acknowledged. First, the sample size was relatively modest, particularly in the control group, which may limit the generalizability of the results. Moreover, the control group consisted of healthy volunteers recruited from a blood donation center. Although all donors were medically screened and confirmed to be in good health, detailed information on their lifestyle and medical background (including physical activity, smoking, alcohol use, and medication history) was not available. Consequently, future research might benefit from using baseline blood samples collected from soldiers before the onset of SERE training—during the theoretical instruction phase—as a more representative reference for physiological comparison. Second, the study was conducted in a specific population of trained male soldiers participating in a standardized SERE (Survival, Evasion, Resistance, and Escape) program. While this may limit the generalizability of the findings to the general population, it also provides a unique and well-controlled human model for studying the neuroendocrine and molecular mechanisms of acute stress adaptation under real-world conditions. Another limitation concerns the study design, which included only two sampling points: during peak SERE stress and after 28 days of recovery. While this approach allowed for a clear contrast between acute and recovery phases, it precluded a finer-grained assessment of intermediate hormonal fluctuations across the recovery period. Including additional time points would enhance the temporal resolution of stress adaptation processes. Furthermore, only male participants were included, leaving potential sex-specific differences in catecholaminergic stress responses unexplored. Expanding future studies to include female soldiers and larger, more diverse cohorts would help provide a broader understanding of neuroendocrine adaptation to extreme stress. Finally, as with other military field studies, logistical and ethical constraints limited access to participants and restricted the scope of physiological and behavioral measurements. Despite these challenges, the current findings provide novel insights into neurochemical adaptation following SERE training and underscore the need for longitudinal designs that track cumulative exposure to high-stress environments.

## 4. Materials and Methods

### 4.1. Participants

The study cohort comprised 47 male operators from an elite special forces unit who underwent SERE training. The soldiers had a mean age of 31.53 ± 3.83 years, a mean BMI of 25.02 ± 1.58, and an average of 9.3 ± 4.80 years of military service, including 3.74 ± 1.61 years in special forces. A comparison group of 17 healthy volunteers was recruited from a local blood donation center (mean age 27.06 ± 4.63 years, BMI 27.32 ± 2.86). All blood donors were male and completed a standard pre-donation health questionnaire that included questions on smoking, alcohol use, chronic diseases, and medication intake, primarily for medical screening purposes.

Inclusion criteria for the study were service in a military special forces unit, participation in and completion of the SERE course, and provision of written informed consent. Exclusion criteria included inability to complete SERE, presence of chronic medical conditions, or refusal to consent.

### 4.2. SERE Training Protocol and Stress Exposure

The SERE course, during which this study was conducted, lasted approximately two-three weeks and consisted of three main phases. During the first (classroom) phase (duration of around 5–8 days), participants received theoretical instruction on survival, evasion, resistance, and escape principles. The second phase (duration of around 5–7 days) involved survival training in a natural environment. Soldiers were required to apply theoretical knowledge in the field, including food and water procurement, signaling, shelter construction, fire-making, and navigation. Training occurred in varied environmental conditions, with exposure to both high and low extreme temperatures, sleep deprivation, and caloric restriction. These combined stressors were designed to simulate prolonged field operations under demanding physical and environmental conditions. The third phase (duration of around 7 days) represented a simulated captivity scenario, in which trainees were “captured” and subjected to mock interrogations, isolation, and psychological pressure. This phase was designed to replicate the conditions of real captivity, generating intense acute stress. Blood samples for this study were collected during the most stressful part of this phase, after multiple interrogation sessions and a period of isolation, identified by SERE instructors as the peak stress point of the course.

Throughout all phases of the training, medical personnel were continuously on standby, and all activities were supervised by certified SERE instructors to ensure participant safety.

### 4.3. Blood Sampling and Biochemical Assays

Blood samples were obtained from soldiers at two time points: (1) during the captivity phase identified as maximally stressful, and (2) four weeks after course completion, when participants had resumed normal duties without extreme operational loads. Control group samples were collected during routine blood donation procedures. All blood draws occurred in the morning hours. Samples were incubated at room temperature for at least 30 min, centrifuged at 1500× *g* for 10 min at 4 °C, and serum aliquots were stored at −80 °C until analysis. Concentrations of norepinephrine and dopamine were determined in duplicate using commercially available ELISA kits (Wuhan Fine Biotech Co., Ltd., Wuhan, China) according to the manufacturer’s instructions. Limits of detection were 9.375 pg/mL for norepinephrine and 0.938 ng/mL for dopamine. Intra- and inter-assay coefficients of variation were <8% and <10%, respectively.

**Figure 3:**
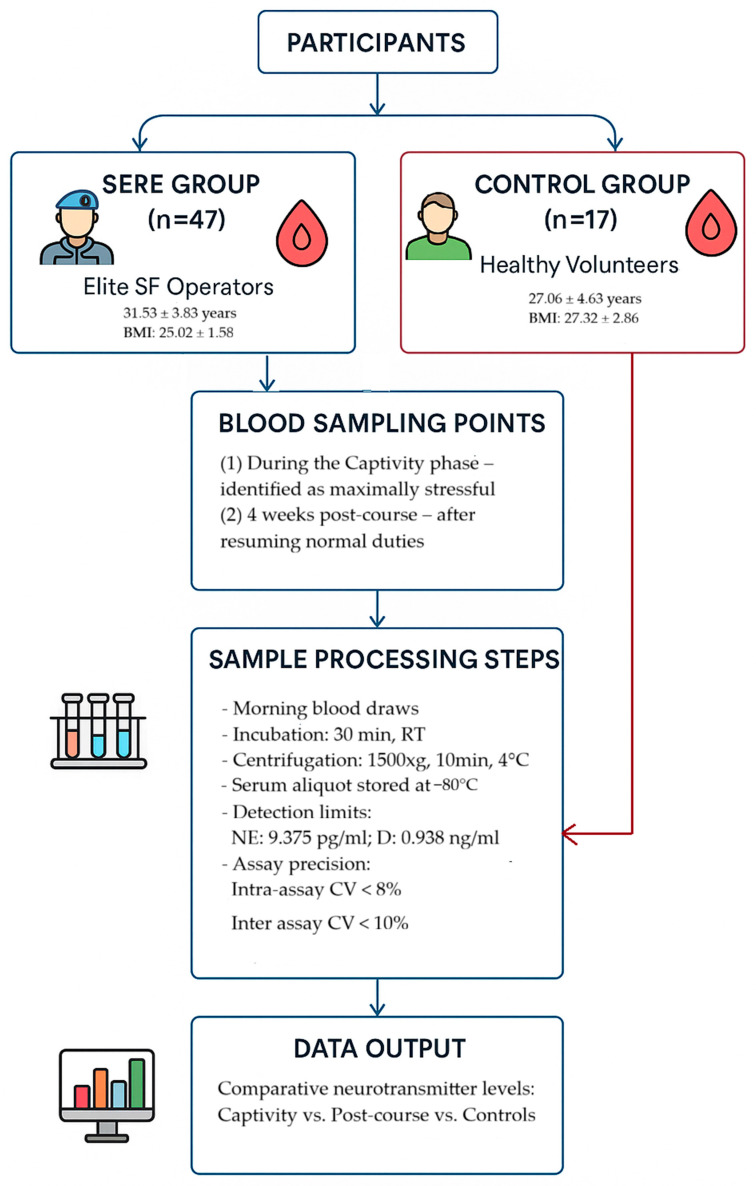
Schematic diagram of the sample processing and analytical workflow.

The sample collection and analysis workflow is illustrated in Figure 3.

### 4.4. Statistical Analysis

Data analyses were conducted using STATISTICA (version 13.3, StatSoft Inc., Tulsa, OK, USA) and jamovi (version 2.4, Sydney, Australia). Results are presented as the mean ± SD or median (interquartile range). The Shapiro–Wilk test was used to assess distributional assumptions. Between-group differences were analyzed using Kruskal–Wallis ANOVA with post hoc testing, and within-subject comparisons were evaluated using Wilcoxon signed-rank tests. Correlations were calculated using Spearman’s rank method. A two-tailed *p*-value < 0.05 was considered statistically significant.

### 4.5. Ethical Approval

The study protocol was reviewed and approved by the Bioethics Committee of the University of Rzeszów (approval no. 3/01/2021, issued 28 January 2021). All procedures adhered to the principles of the Declaration of Helsinki and its later amendments. Written informed consent was obtained from all participants.

## 5. Conclusions

In conclusion, the present study provides new insights into catecholaminergic adaptation to extreme psychophysiological stress. The observed increase in norepinephrine during SERE training and its subsequent decline after one month, accompanied by a modest but persistent elevation in dopamine, suggest a coordinated neuroendocrine recalibration that supports recovery and resilience following intense stress exposure. These findings highlight the importance of assessing both immediate and delayed neurochemical responses to better understand the mechanisms underlying long-term adaptation in military personnel. Future research should expand on these results by incorporating additional time points, behavioral assessments, and multidimensional biomarkers to further elucidate the interplay between neuroendocrine regulation, cognitive recovery, and operational performance.

## Data Availability

The original contributions presented in this study are included in the article/Supplementary Material. Further inquiries can be directed to the corresponding author.

## Supplementary Materials

The following supporting information can be downloaded at: https://www.mdpi.com/article/10.3390/ijms262211012/s1.
