# Supplementary material for: Catecholaminergic Adaptation to Extreme Military Stress: Norepinephrine and Dopamine Responses During and After SERE Training
Source: Int J Mol Sci. 2025 Nov 14;26(22):11012. doi: 10.3390/ijms262211012 (PMC12651961; doi:10.3390/ijms262211012)
Supplement: Supplementary file 1 [file ijms-26-11012-s001.zip › S1.pdf]

Application: Tecan i-control | Tecan i-control , 1.10.4.0
Device: infinite 200Pro | Serial number: 1211001057 | Serial number of connected stacker:
Firmware: V_3.37_07/12_Infinite (Jul 20 2012/13.56.47 | MAI, V_3.37_07/12_Infinite (Jul 20 2012/13.56.47)

Date: 29.11.2022
Time: 13:25:53

System USER-KOMPUTER
User User-Komputer\User
Plate: Greiner 96 Flat Bottom Transparent Polystyrol [GRE96ft.pdfx]
Plate-ID (Stacker)

Label: Label1
Mode: Absorbance
Wavelength: 450 nm
Bandwidth: 9 nm
Number of Flashes: 25
Settle Time: 0 ms
Start Time: 2022-11-29 13:25:53

Temperature: 24.1 °C

x	1	2	3	4	5	6	7	8	9	10	11	12
A	0.17399999	0.17800199	1.70120001	1.74032761	1.60500002	1.58931917	1.29560006	1.32539886	1.10800004	1.09717488	1.15859997	1.14728045
B	0.2475	0.2531925	1.32480001	1.35527041	1.48810005	1.50595725	1.49590003	1.53030574	1.24380004	1.25872564	1.39180005	1.40850165
C	0.40040001	0.41201161	1.98870003	2.04637233	1.54960001	1.50760585	1.29799998	1.33564198	1.04159999	1.01337263	1.00419998	1.01417068
D	0.56230003	0.57523293	0.70120001	0.71732761	1.4217	1.4543991	1.06369996	1.08816506	0.82590002	0.84489572	0.50489998	0.51651268
E	0.89399999	0.85823999	1.53410006	1.47273605	1.69140005	1.73030225	1.62759995	1.56249596	1.26499999	1.29409499	1.5431	1.5785913
F	1.11489999	1.08145299	1.63460004	1.61661944	1.32379997	1.30659057	1.24600005	1.23229405	1.55449998	1.53429148	1.03299999	1.02132709
G	1.59519994	1.64784154	1.27030003	1.31221993	1.40450001	1.45084851	1.46580005	1.51417145	1.57280004	1.62470244	1.37440002	1.39226722
H	2.0624001	2.12427211	1.48599994	1.53057994	1.45749998	1.50122498	1.30369997	1.34281097	1.28999996	1.32869996	1.34519994	1.38555594

Application: Tecan i-control | Tecan i-control , 1.10.4.0
Device: infinite 200Pro | Serial number: 1211001057 | Serial number of connected stacker:

Firmware: V_3.37_07/12_Infinite (Jul 20 2012/13.56.47) MAI, V_3.37_07/12_Infinite (Jul 20 2012/13.56.47)

Date: 29.11.2022
Time: 13:29:50

System USER-KOMPUTER
User User-Komputer\User
Plate Greiner 96 Flat Bottom Transparent Polystyrol [GRE96ft.pdfx]
Plate-ID (Stacker)

Label: Label1

Mode	Absorbance	
Wavelength	450	nm
Bandwidth	9	nm
Number of Flashes	25	
Settle Time	0	ms

Start Time: 2022-11-29 13:29:50

Temperature: 24.1 °C

<>	1	2	3	4	5	6	7	8	9	10	11	12
A	0.17622429	0.174464337	1.2105	1.226236502	1.30659997	1.323585774	1.30429995	1.29155694	1.15470004	1.10800004	1.10070002	1.089946182
B	0.25069172	0.248215638	1.09930003	1.112821417	1.30509996	1.321152694	1.30280006	1.31843366	1.16470003	1.243800044	1.27999997	1.295359971
C	0.40385378	0.395857478	1.30519998	1.33130398	1.06939995	1.082232752	1.42659998	1.38793912	0.87449998	1.041599989	1.0201	0.992455288
D	0.58271096	0.590286197	0.66259998	0.67783978	0.75139999	0.768682194	1.08650005	1.11148955	0.755	0.825900018	0.70230001	0.718452912
E	0.83249279	0.80751801	1.29999995	1.247999954	1.31289995	1.260383949	1.3513	1.382379901	1.33510005	1.264999986	1.08749998	1.112512476
F	1.09215938	1.102971756	1.26100004	1.248390037	1.40610003	1.392039034	1.13230002	1.117580119	1.41600001	1.554499984	1.01380002	1.000620625
G	1.6763492	1.70535004	1.14890003	1.186813733	1.17400002	1.212742026	1.28079998	1.323066384	1.24790001	1.57280004	1.01849997	1.052110469
H	2.15188764	2.179862182	1.20079994	1.23682394	1.14129996	1.175538962	1.12759995	1.161427953	1.17540002	1.289999962	0.84909999	0.874572994

Application: Tecan i-control Tecan i-control , 1.10.4.0
Device: infinite 200Pro Serial number: 1211001057 Serial number of connected stacker:
Firmware: V_3.37_07/12_Infinite (Jul 20 2012/13.56.47) MAI, V_3.37_07/12_Infinite (Jul 20 2012/13.56.47)

Date: 29.11.2022
Time: 13:33:32

System	USER-KOMPUTER
User	User-Komputer\User
Plate	Greiner 96 Flat Bottom Transparent Polystyrol [GRE96ft.pdfx]
Plate-ID (Stacker)	

Label: Label1

Mode	Absorbance	
Wavelength	450	nm
Bandwidth	9	nm
Number of Flashes	25	
Settle Time	0	ms

Start Time: 2022-11-29 13:33:32

Temperature: 24.1 °C

<>	1	2	3	4	5	6	7	8	9	10	11	12
A	0.178233397	0.180550432	1.55229998	1.536978776	1.30633865	1.32332106	1.28603975	1.25565166	1.11463195	1.05884916	1.04124021	1.06265393
B	0.25069172	0.253775228	1.28680003	1.302627667	1.30183721	1.323795	1.28156442	1.32436661	1.16190475	1.24115075	1.27761917	1.29190136
C	0.407973899	0.412869586	0.98000002	0.991760019	1.06426683	1.08677813	1.40006522	1.43901528	0.90064753	1.08336815	1.07141103	1.01200666
D	0.588463284	0.60199794	0.58420002	0.597636625	0.74606505	0.77360176	1.0637922	1.18829348	0.80196099	0.89389637	0.77425767	0.74867823
E	0.823910394	0.81567129	1.49619997	1.436351967	1.30055869	1.27122325	1.31994984	1.39639723	1.46126701	1.28075683	1.25564922	1.15188207
F	1.070638463	1.059932079	0.86989999	0.861200988	1.38964866	1.40707306	1.10342637	1.15524257	1.43395489	1.58042749	1.03476338	1.00881671
G	1.669757833	1.708162263	1.08159995	1.117292749								
H	2.188000269	2.123016661	0.83429998	0.859328981								
