# Supplementary material for: Catecholaminergic Adaptation to Extreme Military Stress: Norepinephrine and Dopamine Responses During and After SERE Training
Source: Int J Mol Sci. 2025 Nov 14;26(22):11012. doi: 10.3390/ijms262211012 (PMC12651961; doi:10.3390/ijms262211012)
Supplement: Supplementary file 1 [file ijms-26-11012-s001.zip › S2.pdf]

Application: Tecan i-control | Tecan i-control , 1.10.4.0
Device: infinite 200Pro | Serial number: 1211001057 | Serial number of connected stacker:
Firmware: V_3.37_07/12_Infinite (Jul 20 2012/13.56.47) | MAI, V_3.37_07/12_Infinite (Jul 20 2012/13.56.47)

Date: 01.12.2022
Time: 14:15:33

System: USER-KOMPUTER
User: User-Komputer\User
Plate: Greiner 96 Flat Bottom Transparent Polystyrol [GRE96ft.pdfx]
Plate-ID (Stacker)

Label: Label1

Mode	Absorbance	
Wavelength	470	nm
Bandwidth	9	nm
Number of Flashes	25	
Settle Time	0	ms

Start Time: 2022-12-01 14:15:33

Temperature: 20.3 °C

<>	1	2	3	4	5	6	7	8	9	10	11	12
A	0.52429998	0.53478598	2.09144001	2.11862873	1.91984005	1.94479797	1.93376007	1.91486723	1.92472	1.94974136	2.04328003	2.09027547
B	0.88209999	0.90856299	2.04160004	2.06671172	2.04391994	2.06906016	2.05799999	2.08269599	2.02168007	2.04654673	2.17224007	2.22220159
C	1.42159998	1.39316798	1.91000004	1.93292004	2.05552006	2.09663046	1.89104004	1.83979285	1.94080009	1.9796161	2.04167995	2.10088867
D	1.92910004	2.02555504	2.05984001	2.10721633	2.04503994	2.09207586	2.12287998	2.17170622	2.03544006	2.08225518	2.13600006	2.18512806
E	2.36080003	2.40801603	2.01720009	1.93651208	2.04416008	1.96239368	1.78176003	1.82274051	1.93304005	1.85571844	1.94880009	1.87084808
F	2.48119998	2.53082398	2.07695999	2.05619039	1.99391994	1.97398075	1.63208008	1.61086304	1.74263992	1.72521352	1.95167999	1.93021151
G	2.52749991	2.67914991	1.95408001	2.01856465	1.51943998	1.5695815	1.85736008	1.91865296	1.82632008	1.88658864	1.87663994	1.93856906
H	2.36339998	2.41066798	2.06896	2.1310288	2.00063992	2.06065911	1.75872002	1.81148162	1.68064003	1.73105923	1.97336006	2.03256086

Application: Tecan i-control Tecan i-control , 1.10.4.0
Device: infinite 200Pro Serial number: 1211001057 Serial number of connected stacker:
Firmware: V_3.37_07/12_Infinite (Jul 20 2012/13.56.47) MAI, V_3.37_07/12_Infinite (Jul 20 2012/13.56.47)

Date: 01.12.2022
Time: 14:18:13

System USER-KOMPUTER
User User-Komputer\User
Plate Greiner 96 Flat Bottom Transparent Polystyrol [GRE96ft.pdfx]
Plate-ID (Stacker)

Label: Label1
Mode Absorbance
Wavelength 470 nm
Bandwidth 9 nm
Number of Flashes 25
Settle Time 0 ms
Start Time: 2022-12-01 14:18:13

<>	1	2	3	4	5	6	7	8	9	10	11	12
A	0.52429998	0.53478598	1.94855995	1.92952252	1.96343994	1.98896466	1.85688	1.88101944	1.84487991	1.82685544	1.89624004	1.92089116
B	0.88209999	0.90856299	1.98607998	2.00991294	2.06152	2.0868767	2.00319996	2.02783932	1.62959995	1.64915515	2.08887997	2.11457319
C	1.42159998	1.39316798	2.02567997	1.97078404	2.03264008	2.05703176	2.05879993	2.09997593	1.87847996	1.82757315	1.92087994	1.95929753
D	1.92910004	2.02555504	2.06632004	2.1138454	2.09223995	2.14036147	2.08808002	2.13610587	1.98344002	2.02905914	1.94447994	1.98920298
E	2.36080003	2.40801603	1.82511997	1.86709773	1.98600006	1.90656006	1.88880005	1.81324805	1.88416004	1.92749572	1.89967995	1.82369275
F	2.48119998	2.53082398	1.97103996	1.94541644	1.82936001	1.81106641	1.91608009	1.89691929	1.89792004	1.87324708	1.87992001	1.86112081
G	2.52749991	2.67914991	1.85911999	1.92047095	1.91616001	1.97939329	1.76888008	1.82725312	1.80048008	1.85989592	1.67880001	1.73420041
H	2.36339998	2.41066798	1.80279999	1.85688399	1.81856003	1.87311683	1.81560001	1.87006801	1.72479992	1.77654392	1.88015995	1.93656475

Application: Tecan i-control Tecan i-control , 1.10.4.0
Device: infinite 200Pro Serial number: 1211001057 Serial number of connected stacker:

Firmware: V_3.37_07/12_Infinite (Jul 20 2012/13.56.47)   MAI, V_3.37_07/12_Infinite (Jul 20 2012/13.56.47)

Date: 01.12.2022
Time: 14:24:27

System USER-KOMPUTER
User User-Komputer\User
Plate Greiner 96 Flat Bottom Transparent Polystyrol [GRE96ft.pdfx]
Plate-ID (Stacker)

Label: Label1
Mode Absorbance
Wavelength 470 nm
Bandwidth 9 nm
Number of Flashes 25
Settle Time 0 ms
Start Time: 2022-12-01 14:24:27

<>	1	2	3	4	5	6	7	8	9	10	11	12
A	0.52429998	0.53478598	1.88560009	1.86698922	1.94041193	2.04127162	1.99732045	1.97613268	1.98735958	1.79515059	1.817114	1.66741557
B	0.88209999	0.90856299	1.76303997	1.78472536	2.03035146	2.14027206	2.03263902	2.10165503	2.08306863	1.97921529	1.94086321	1.82383741
C	1.42159998	1.39316798	1.71023998	1.73076286	1.92376145	1.95698505	1.79117274	2.05908486	1.94527517	1.95971486	1.99523385	1.51543734
D	1.92910004	2.02555504	1.82872009	1.87078065	1.73026718	1.96417074	1.92984523	1.95576878	2.03526028	2.05417579	2.07253661	1.7446752
E	2.36080003	2.40801603	1.48615999	1.42671359	1.80915267	1.89277904	1.8159884	1.93558987	1.93603925	1.82290686	1.93837607	1.77316416
F	2.48119998	2.53082398	1.67423992	1.65749752	1.66097654	1.99467235	1.75682859	1.8798455	1.83862182	1.75356786	1.9190379	1.58713462
G	2.52749991	2.67914991	1.71256008	1.76907456								
H	2.36339998	2.41066798	1.57424002	1.62146722								
